# An Introduction to the Toxins Special Issue on the Adenylate Cyclase Toxin

**DOI:** 10.3390/toxins10100386

**Published:** 2018-09-24

**Authors:** Alexandre Chenal

**Affiliations:** Institut Pasteur, Biochemistry of Macromolecular Interactions Unit, UMR CNRS 3528, Structural Biology and Chemistry Department, 28 rue du Docteur Roux, 75724 Paris CEDEX 15, France; chenal@pasteur.fr

The adenylate cyclase (CyaA) toxin is produced by *Bordetella pertussis*, the causative agent of whooping cough. The incidence of pertussis is currently increasing, and represents a global public health concern. *Bordetella pertussis*, a Gram-negative bacteria, has been identified by Jules Bordet and Octave Gengou who initially described “Le microbe de la coqueluche” in an article published in the *Annales de l’Institut Pasteur* in 1906. During the last decades, multidisciplinary approaches have contributed to improve our knowledge on CyaA, and showed that this toxin plays a crucial role in the early stages of respiratory tract colonization by disrupting the host immune response. CyaA is a 1706-residue-long, multi-domain, and bifunctional toxin containing a hemolysin and a calmodulin-activated adenylate cyclase producing supraphysiological levels of cAMP in host cells. This toxin is a unique well-characterized bacterial toxin able to translocate its catalytic domain directly across the plasma membrane of target cells. The molecular mechanism by which CyaA intoxicates host cells remains, however, largely unknown. Recent advances worldwide open new perspectives for both basic sciences and CyaA-based biotechnological applications, such as antigen delivery vehicles and CyaA-containing pertussis vaccines. These various aspects are discussed in this Special Issue of Toxins on the adenylate cyclase toxin.

The first review from Nicole Guiso provides us with an overview on the properties of CyaA toxins produced by the bacteria from the genus *Bordetella* [1]. Due to the crucial contributions of the CyaA toxin at the early stages of whooping cough and its low variability, Nicole Guiso further discusses the relevance of CyaA as a vaccine antigen against whooping cough. 

Pertussis colonization of the host respiratory tract launches the primary innate immune defense. In their review, Giorgio Fedele and colleagues describe how CyaA is actively involved in the subversion of host immune responses by intoxicating a vast array of target cells [2]. In particular, the authors focus on the massive amounts of cAMP produced by CyaA in dendritic cells, alveolar macrophages, and neutrophils, leading to various effects and dramatically altering the host immune response. 

The ability of CyaA to deliver its catalytic domain into antigen presenting cells, such as in dendritic cells, has been exploited to engineer CyaA-based non-replicating recombinant proteins in which antigens are genetically inserted into the detoxified CyaA catalytic domain. Daniel Ladant and myself review these biotechnological applications he initiated in collaboration with Claude Leclerc in the early 1990s [3]. We illustrate the use of CyaA-based recombinant vaccines with data from clinical trials.

This Special Issue then focuses on the molecular processes involved in CyaA biogenesis, membrane translocation, and the effects of cAMP intoxication. CyaA is produced as a protoxin, proCyaA, which is acylated in the bacteria before its secretion. Acylation is crucial for CyaA activation, i.e., to gain the ability to translocate its catalytic domain into target host cells. In a research article, Valérie Bouchez and colleagues characterize the nature of the acyl chains of CyaA toxins from various *Bordetella* isolates using state-of-the-art mass spectrometry methods [4]. The authors further analyzed the cytotoxicity of CyaA toxins against macrophages and showed that only CyaA from pertussis exhibits a cytotoxic activity against macrophages. 

Jakub Novak et al. and Helena Ostolaza et al. provide two complementary reviews describing the molecular basis of CyaA biogenesis and translocation. Novak and colleagues further highlight the diverse effects of CyaA on host phagocytes [5], while the Ostolaza group provides a detailed description of the potential molecular processes leading to CyaA translocation across the plasma membrane of target cells [6]. 

In a research article, Alexis Voegele and colleagues present new results on a membrane-active peptide derived from the CyaA translocation region, which is required for the delivery of the catalytic domain into host cytosol. They show that the arginine residues from the segment 454–484 from CyaA are involved in the successive steps of membrane interaction, folding, and permeabilization [7]. The authors propose that this region induces a local destabilization of the membrane, decreasing the energy required to translocate the catalytic domain across the plasma membrane. 

Kurehong and collaborators report the effect on the pore-forming activity of point mutations of two residues (Q574 and E581) from the hydrophobic region of CyaA. They show that mutations to positively charged residues, which mimic the local positive valence as observed in hemolytic RTX proteins, increase the pore forming activity of CyaA [8]. Hence, compared to the efficiency of other bacterial hemolysins, the hemolytic activity of CyaA is rather low; however, this loss of hemolytic activity might be explained by the fact that the main functional property of CyaA is to intoxicate cells by translocating its catalytic domain into host cells and to induce a cascade of events disrupting host immune responses. 

These last decades, intensive research activities have been dedicated to investigate the activation of the CyaA enzymatic domain. Recently, we showed that the catalytic domain contains a large region of structural disorder [9], and is poorly active in the absence of calmodulin, while calmodulin binding induces local folding, leading to the stabilization of the enzymatic core and the production of massive amounts of cAMP at the expense of ATP. Christian Johns and Natosha Finley report that the inactivation of calcium binding at Site 1 of calmodulin affects the stability of the N-terminal region of calmodulin and, consequently, its interaction with the catalytic domain of CyaA [10]. In the same topic, Thérèse Malliavin used molecular dynamics to highlight the contributions of several regions from both calmodulin and the CyaA catalytic domain to the formation of the active enzymatic complex [11]. 

Finally, Beyza Bulutoglu and Scott Banta review the works they have done using the C-terminal RTX domain of CyaA to engineer calcium-dependent nanotools they developed in the fields of bioseparation, hydrogel catalysis, and molecular recognition [12]. These applications are based on the remarkable properties of the RTX motifs, which are intrinsically disordered in the absence of calcium and undergo a disorder-to-order transition upon calcium binding [13,14]. 

Taken together, CyaA is intensively investigated to provide new insights into its intoxication process and how it disrupts host immune responses. As illustrated in the reviews and research articles of this Special Issue, methodologies from fundamental sciences applied to CyaA are crucial to provide a better understanding of the toxin and also to develop CyaA-based biotechnologies and vaccines. 
